# Subgroup disproportionality analysis of dementia-related adverse events with sacubitril/valsartan across geographical regions

**DOI:** 10.1038/s41598-024-67050-5

**Published:** 2024-09-03

**Authors:** Seong Kyung Kim, Myeong Gyu Kim

**Affiliations:** College of Pharmacy and Graduate School of Pharmaceutical Sciences, Ewha Womans University, 52 Ewhayeodae-gil, Seodaemun-gu, Seoul, 03760 Korea

**Keywords:** Sacubitril/valsartan, Dementia, FDA adverse event reporting system, Geographic location, Cardiology, Neurology

## Abstract

This study aimed to evaluate the association between sacubitril/valsartan and dementia-related adverse events (AEs) in geographical subpopulations using subgroup disproportionality analysis. Cases from the FDA adverse event reporting system involving patients aged 60 or older with sacubitril/valsartan or angiotensin receptor blockers (ARBs) were analyzed. The adjusted reporting odds ratios (RORs) for dementia-related AEs were calculated for each continent. A total of 61,518 AEs associated with sacubitril/valsartan or ARBs were identified. Among these, 1441 were dementia-related AEs. In Asia, Europe, and Africa, the reporting risk of dementia-related AEs associated with sacubitril/valsartan was lower compared to ARBs (adjusted ROR, 0.57 [95% CI 0.31–1.01]; adjusted ROR, 0.89 [95% CI 0.69–1.14]; adjusted ROR, 0.40 [95% CI 0.27–0.61], respectively). In Latin America and Oceania, the reporting risk of dementia-related AEs associated with sacubitril/valsartan was similar to that associated with ARBs (adjusted ROR, 1.04 [95% CI 0.75–1.44]; adjusted ROR, 1.02 [95% CI 0.31–3.37], respectively). On the contrary, in North America, the reporting risk associated with sacubitril/valsartan was higher compared to ARBs (adjusted ROR, 1.29 [95% CI 1.10–1.53]). Although the ROR value did not meet the criteria for signal detection, the significantly greater than 1 ROR observed in North America suggests that caution may be warranted regarding potential dementia-related adverse events associated with sacubitril/valsartan.

## Introduction

Sacubitril/valsartan, the first-in-class angiotensin receptor-neprilysin inhibitor, combines sacubitril, a prodrug for the neprilysin inhibitor (sacubitrilat), with valsartan, an angiotensin receptor blocker (ARB)^1^. In 2015, the U.S. FDA (Food and Drug Administration) approved sacubitril/valsartan for treating chronic heart failure (NYHA [New York Heart Association] Class II-IV) with a reduced ejection fraction^1^. Sacubitril inhibits neprilysin, an enzyme responsible for metabolizing various vasoactive peptides, including natriuretic peptides, bradykinin, substance P, and angiotensin II^2^. Among these, natriuretic peptides, including atrial natriuretic peptide and brain natriuretic peptide, manifest beneficial impacts on heart failure pathogenesis. These peptides induce augmented natriuresis and diuresis, facilitate vasodilation, exhibit anti-proliferative properties, and mitigate sympathetic activity^3^.

Despite the potential benefits of neprilysin inhibitors in heart failure treatment, concerns endure because of the wide-ranging effects on diverse physiological functions resulting from the inhibition of neprilysin^4^. Neprilysin plays a pivotal role in degrading amyloid-β, contributing to as much as half of the total amyloid-β clearance in the brain. Numerous in vitro and in vivo experiments have underscored the critical role of neprilysin in Alzheimer’s disease^5–7^. Neprilysin inhibition by sacubitril could lead to the accumulation of amyloid-β, a key pathological feature of Alzheimer’s disease^8^. Upon approval of sacubitril/valsartan by the US Food and Drug Administration (FDA), a randomized trial was mandated to assess its impact, relative to valsartan, on cognitive function in patients with chronic heart failure^9^. The PERSPECTIVE trial was conducted to compare the effects of sacubitril/valsartan versus valsartan on cognitive function in patients aged 60 and older with heart failure and mildly reduced or preserved ejection fraction^10^. There was no significant difference in the change of Global Cognitive Composite Score (GCCS) from baseline to 3 years between patients who received sacubitril/valsartan and those treated with valsartan^10^. The adjusted least-squares mean change in GCCS was − 0.0180 (*p* = 0.74)^10^. Additionally, there was no significant difference in the deposition of amyloid-β measured by positron emission tomography between the two groups^10^.

On the contrary, in a retrospective cohort study, the composite outcome of cognitive decline, dementia, and Alzheimer’s disease was significantly lower in the sacubitril/valsartan group compared to the angiotensin-converting enzyme inhibitors (ACEI)/ARB group (3-year incidence, 10.7% vs 15.0%; hazard ratio [HR] 0.69; *p* < 0.001)^11^. Improvement in cerebral perfusion and reduction in inflammatory cytokines due to sacubitril/valsartan was suggested as reasons for the reduction in cognitive dysfunction in heart failure patients^11^. According to subgroup analysis, the composite outcome was significantly lower in both White (HR 0.75; *p* < 0.001) and Black patients (HR 0.60; *p* < 0.001) in the sacubitril/valsartan group compared to the ACEI/ARB group^11^.

However, the 3-year incidence was more frequent in White patients at 11.8% compared to 8.8% in Black patients^11^. Furthermore, the ARB component within sacubitril/valsartan is known to have a lower risk of cognitive decline compared to ACEIs^12^. The inclusion of ACEIs in the control group could potentially have underestimated the risk of cognitive dysfunction associated with sacubitril/valsartan. Therefore, there is a need to investigate the correlation between dementia-related adverse events (AEs) and sacubitril/valsartan stratified by race or region. Disparities in dementia-related AEs across different racial or regional groups may stem from genetic and environmental influences. For instance, polymorphisms in the membrane metalloendopeptidase (MME) gene encoding neprilysin have been associated with Alzheimer’s disease and could be related to the risk of dementia with sacubitril/valsartan^13–16^.

Dementia-related AEs, given their delayed onset and low incidence rate, have been evaluated using real-world pharmacovigilance databases rather than clinical trials. The prominent database, FDA Adverse Event Reporting System (FAERS), contains reports submitted to the FDA. While the majority of FAERS reports originate from the United States, there are also submissions from foreign sources^17^. The occurrence country field in the FAERS dataset enables subgroup analysis based on regions. Subgroup disproportionality analysis allows for the identification of specific patient subpopulations at increased risk for AEs^18,19^. While previous studies have analyzed the relationship between sacubitril/valsartan and dementia-related AEs using FAERS data, regional analysis has not been conducted^8,20^. This study aimed to evaluate the association between sacubitril/valsartan and dementia-related AEs in geographical subpopulations using subgroup disproportionality analysis.

## Methods

### Data collection

The FAERS quarterly data files (https://open.fda.gov/data/faers/) from the third quarter of 2015 through the fourth quarter of 2022 were used, which include the approval date of sacubitril/valsartan in July 2015. The quarterly data files contain demographic (DEMO file), drug (DRUG file), and reaction (REAC file) information compiled from the reports. All files are linked through “PRIMARYID”, a unique number for identifying a FAERS report. The study protocol was exempted from review by the institutional review board of Ewha Womans University (ewha-202308-0021-01).

### Data processing

The quarterly data were integrated into one file; duplicate and follow-up reports for individual patients were removed. Then, only reports with sacubitril/valsartan or an ARB as the primary suspected drug, and those reporting on patients aged 60 years or older were retained. Lastly, the REAC file was merged to create drug–AE pairs.

Descriptive analysis was conducted for age, sex, occurrence country (categorized by continent), reporting year, and reporter type. Age data were analyzed using medians and quartiles based on the results of the Kolmogorov–Smirnov test, and differences between sacubitril/valsartan and ARBs were examined using the Mann–Whitney U test. The remaining data were analyzed using frequency analysis and chi-square tests.

### Disproportionality analysis

The comparison of dementia-related AEs between sacubitril/valsartan and ARBs was conducted using disproportionality analysis. The drug–AE pairs were presented in a 2 × 2 contingency table (Table  S1). From this table, the ROR with 95% confidence interval (95% CI) was calculated as (A/C)/(B/D). ROR values greater than 1 were considered indicative of an increase in the reporting of AEs. Dementia-related AEs were defined by using SMQs (Standardized MedDRA Queries) with preferred terms related to dementia-related AEs in MedDRA 26.0^21^. Previous studies have evaluated both broad and narrow definitions (SMQ) for dementia-related AEs^8,20^. Due to the nature of spontaneous reporting, which can be submitted by non-experts, this study used the broad SMQ rather than a narrower dementia-specific definition. The precise preferred terms are documented in Table S2. The list of ARBs contains candesartan, eprosartan, irbesartan, losartan, olmesartan, telmisartan, and valsartan.

The ROR was analyzed by continent: Asia, Africa, Europe, North America, Latin America, and Oceania. The “occurrence country” in the FAERS database was categorized into six geographic regions using the standard country codes of the United Nations^22^. To control for confounding factors such as age, sex, and reporter type, adjusted ROR was calculated using multiple logistic regression. Age was stratified into three groups: 60–69 years, 70–79 years, and 80 years and older. In the sensitivity analysis, only reports with indication of heart failure were extracted for the disproportionality analysis. The term corresponding to heart failure is documented in Table S3. All data processing and analysis were performed using SAS, version 9.4 (SAS Institute Inc., Cary, NC, USA).

## Results

### Data characteristics

**Figure 1:**
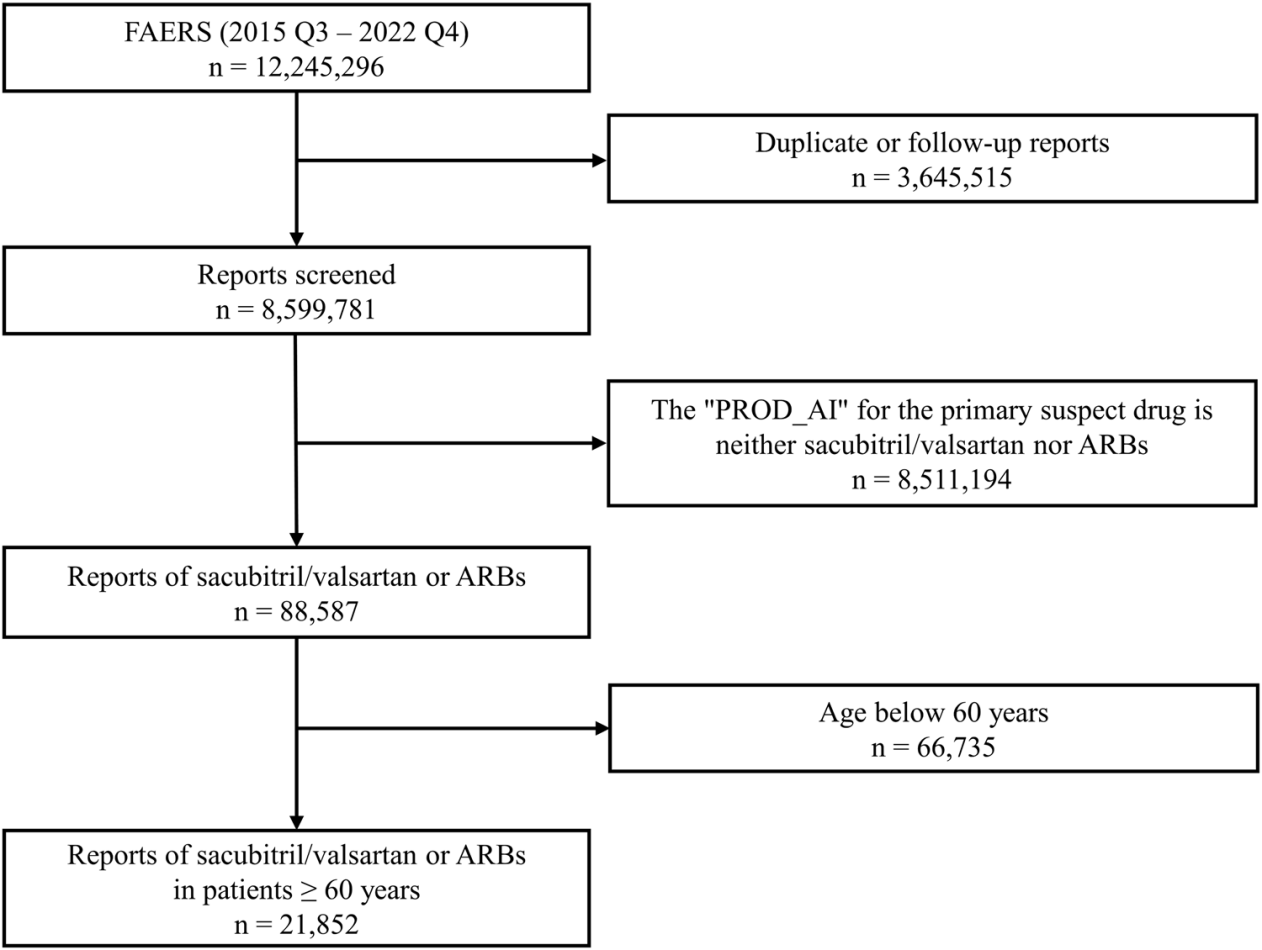
Flow diagram of the report selection process. Abbreviations: AE, adverse event; ARB, angiotensin receptor blocker; FAERS, U.S. Food and Drug Administration adverse event reporting system; PROD_AI, Product active ingredient.

A flow diagram of report selection is shown in Fig. 1. Overall, we identified 61,518 AEs associated with sacubitril/valsartan or ARBs in patients aged 60 and above. Among these, 1441 dementia-related AEs were identified for either sacubitril/valsartan or ARBs. Specifically, sacubitril/valsartan was associated with 544 cases of dementia. The characteristics of the reports included in the study are presented in Tables 1 and S4.

**Table 1:** Characteristics of adverse event reports with sacubitril/valsartan and ARBs.

Characteristics	Total (n = 21,852)	Sacubitril/valsartan (n = 10,733)	ARBs (n = 11,119)	*p*-value
Age (year), median (Q1–Q3)	73 (66–80)	72 (66–80)	73 (67–81)	< 0.0001
Sex, n (%)				< 0.0001
Male	11,592 (53.0)	6930 (64.6)	4662 (41.9)	
Female	9795 (44.8)	3453 (32.2)	6342 (57.0)	
Missing	453 (2.1)	350 (3.3)	103 (1.0)	
Occurrence country, n (%)				< 0.0001
Asia	2355 (10.8)	1754 (16.3)	601 (5.4)	
Africa	410 (1.9)	309 (2.9)	101 (0.9)	
Europe	7249 (33.2)	1495 (13.9)	5754 (51.7)	
North America	9465 (43.3)	6057 (56.4)	3408 (30.7)	
Latin America	1835 (8.4)	978 (9.1)	857 (7.7)	
Oceania	195 (0.9)	110 (1.0)	85 (0.8)	
Missing	343 (1.6)	30 (0.3)	313 (2.8)	
Reporter, n (%)				< 0.0001
Physician	5389 (24.7)	2486 (23.2)	2903 (26.1)	
Pharmacist	1812 (8.3)	255 (2.4)	1557 (14.0)	
Other health-professional	4761 (21.8)	1945 (18.1)	2816 (25.3)	
Consumer/lawyer	9551 (43.7)	6032 (56.2)	3519 (31.6)	
Missing	339 (1.6)	15 (0.1)	324 (2.9)	
Reported year, n (%)				< 0.0001
2015	645 (3.0)	125 (1.2)	520 (4.7)	
2016	1478 (6.8)	589 (5.5)	889 (8.0)	
2017	2069 (9.5)	1225 (11.4)	844 (7.6)	
2018	3301 (15.1)	1448 (13.5)	1853 (16.7)	
2019	4365 (20.0)	1421 (13.2)	2944 (26.5)	
2020	4104 (18.8)	2442 (22.8)	1662 (14.9)	
2021	3086 (14.1)	1852 (17.3)	1234 (11.1)	
2022	2758 (12.6)	1628 (15.2)	1130 (10.2)	
Missing	46 (0.2)	3 (0.03)	43 (0.4)	

*ARB* angiotensin receptor blocker.

### Disproportionality analysis

The results of the disproportionality analysis are presented in Table 2. The crude ROR was 0.90 (95% CI 0.81–1.01), and the adjusted ROR was 0.97 (95% CI 0.86–1.01) in the overall population. In Asia, Europe, and Africa, the reporting risk of dementia-related AEs associated with sacubitril/valsartan was lower compared to ARBs. The adjusted ROR was 0.57 (95% CI 0.31–1.01) in Asia, 0.89 (95% CI 0.69–1.14) in Europe, and 0.40 (95% CI 0.27–0.61) in Africa. In Latin America and Oceania, the reporting risk of dementia-related AEs associated with sacubitril/valsartan was similar to that associated with ARBs. The adjusted ROR was 1.04 (95% CI 0.75–1.44) in Latin America and 1.02 (95% CI 0.31–3.37) in Oceania. On the contrary, in North America, the reporting risk associated with sacubitril/valsartan was higher compared to ARBs, with an adjusted ROR of 1.29 (95% CI 1.10–1.53).

**Table 2:** Number of cases with dementia-related adverse events and reporting odds ratios.

Class	Sacubitril/valsartan	ARBs	Crude ROR (95% CI)	Adjusted ROR (95% CI)^a^
All regions^b^	544/24,648	897/36,870	0.90 (0.81, 1.01)	0.97 (0.86, 1.01)
Asia	24/3879	23/1825	0.49 (0.27, 0.87)*	0.57 (0.31, 1.01)
Africa	8/1251	5/200	0.25 (0.08, 0.78)*	0.40 (0.27, 0.61)*
Europe	83/4486	470/17,687	0.69 (0.55, 0.87)*	0.89 (0.69, 1.14)
North America	333/11,777	284/12,979	1.30 (1.11, 1.53)*	1.29 (1.10, 1.53)*
Latin America	88/2832	84/2932	1.08 (0.80, 1.47)	1.04 (0.75, 1.44)
Oceania	6/317	13/378	0.54 (0.20, 1.44)	1.02 (0.31, 3.37)

*ARB* angiotensin receptor blocker, *CI* confidence interval, *ROR* reporting odds ratio.

*Statistically significant (*p* < 0.05).

^a^Adjusted for age, sex, and reporter.

^b^Including cases where occurrence country is missing.

### Sensitivity analysis

Table 3 presents the results of the analysis focusing on reports specifying heart failure as the indication. In the sensitivity analysis, the number of AEs involving sacubitril/valsartan decreased from 24,648 to 13,976. Among these, dementia-related AEs decreased from 544 to 255. The number of AEs where ARBs were the primary suspected drug decreased from 36,870 to 1607. Among these, dementia-related AEs decreased from 879 to 35. The reporting risk of dementia-related AEs associated with sacubitril/valsartan was consistent with the overall analysis (crude ROR, 0.83 [95% CI 0.58–1.19]; adjusted ROR, 0.92 [95% CI 0.62–1.35]).

**Table 3:** Number of cases with dementia-related adverse events and reporting odds ratios in HF patients.

Class	Sacubitril/valsartan	ARBs	Crude ROR (95% CI)	Adjusted ROR (95% CI)^a^
All regions^b^	255/13,976	35/1607	0.83 (0.58, 1.19)	0.92 (0.62, 1.35)
Asia	16/3272	0/164	NA	NA
Africa	7/1159	3/41	0.08 (0.02, 0.31)*	1.38 (0.03, 56.58)
Europe	50/2659	20/824	0.77 (0.46, 1.30)	0.76 (0.43, 1.34)
North America	142/5335	8/441	1.48 (0.72, 3.04)	1.85 (0.81, 4.26)
Latin America	38/1350	3/76	0.70 (0.21, 2.33)	0.73 (0.21, 2.52)
Oceania	0/115	0/16	NA	NA

*ARB* angiotensin receptor blocker, *CI* confidence interval, *NA* not available, *ROR* reporting odds ratio.

*Statistically significant (*p* < 0.05).

^a^Adjusted for age, sex, and reporter.

^b^Including cases where occurrence country is missing.

In Europe and Latin America, there was a tendency towards lower reporting risk. The adjusted ROR was 0.76 (95% CI 0.43–1.34) in Europe and 0.73 (95% CI 0.21–2.52) in Latin America. In Africa and North America, the reporting risk was higher than 1; yet the confidence intervals were broad (adjusted ROR, 1.38 [95% CI 0.03–56.58]; adjusted ROR, 1.85 [95% CI 0.81–4.26], respectively). Due to the reduction in data size in the sensitivity analysis, dementia-related AEs were not reported in Asia and Oceania, making it impossible to calculate the ROR.

## Discussion

Through subgroup disproportionality analysis, we observed a higher reporting risk of dementia-related AEs for sacubitril/valsartan compared to ARBs in North America. The FAERS database does not include information on the number of patients taking the drug, thus rendering it impossible to determine the frequency of AEs. Instead, the ROR is calculated through disproportionality analysis. An ROR greater than 1 indicates that dementia-related AEs are reported more frequently in sacubitril/valsartan compared to other AEs, in contrast to ARBs. When an AE occurs, it may or may not be reported. Assuming that all AEs have the same reporting probability, an ROR greater than 1 can be interpreted as a higher risk of the AE occurring. For signal detection of AEs, typically the ROR is greater than 2, and the confidence interval of ROR does not include 1^23^. The ROR value in North America was not greater than 2, so it did not exceed the threshold for signal detection. However, the adjusted ROR was 1.29, and it was significantly greater than 1. An ROR greater than 1 could suggest that dementia-related AEs associated with sacubitril/valsartan occurred more frequently compared to ARBs in North America. Alternatively, it could indicate that while the frequency may not differ from ARBs, these events were more frequently reported specifically in North America.

There is no evidence suggesting a higher risk of dementia-related AEs in North America; however, a logical genetic explanation is possible. Neprilysin is widely expressed in various tissues, including the brain, where it is found in pyramidal neurons within the neocortex and in cerebral vascular smooth muscle cells^16^. Its role involves the cleavage of amyloid-β; it is a significant enzyme in amyloid-β degradation^24^. Patients with the T allele of rs701109 in the MME gene had significantly higher neprilysin protein levels, which might result in the promotion of amyloid-β degradation^25^. In the 1000 Genomes Project, Americans had the lowest frequency of the T allele of rs701109 in the MME gene at 0.277 compared to other ethnicities^26^.

As mentioned earlier, while the actual frequency of dementia-related AEs may not vary between sacubitril/valsartan and ARBs, there is also a possibility that dementia-related AEs associated with sacubitril/valsartan are more prominently reported in North America. Notoriety bias, defined as an increase in reporting AEs following the issuance of warnings about them^27^, may have contributed to the results. The FDA’s request for verification of sacubitril/valsartan’s impact on cognitive function may have triggered an increase in the reporting of dementia-related AEs compared to other AEs.

There are other factors that may have impacted our findings of continental differences in dementia-related AEs. In the United States, the target blood pressure for treatment is lower than in Europe^28^, and hypotension caused by medications can increase the risk of dementia^29^. Moreover, there are differences in the penetrating capability across the blood–brain barrier among ARBs^30^, and variations also exist in the ARBs commonly used between countries^31^. Additionally, ARBs are employed in conditions beyond heart failure, and variations in the prevalence of conditions for which ARBs are prescribed may exist across different countries. Epidemiologic research on dementia and Alzheimer’s disease suggests that the prevalence is higher in North America than in Asia, Africa, and Latin America^32^. Furthermore, numerous other risk factors for dementia might be involved in regional differences. These include known modifiable risk factors for dementia: less education, physical inactivity, obesity, smoking, excessive alcohol consumption, air pollution, head injury, high blood pressure, diabetes, depression, social isolation, and hearing loss^33^. These factors naturally vary considerably across continents.

Sensitivity analysis focusing exclusively on heart failure patients did not reflect statistically significant findings. The lack of statistical significance may be attributable to the small number of cases. Limiting the analysis to reports specified for heart failure indication introduces bias. Sacubitril/valsartan is currently used exclusively for heart failure, but restricting the indication resulted in a halving of the number of reports. Due to the voluntary reporting nature, there are often many missing values, particularly in the indication field. Therefore, caution is needed in interpreting sensitivity analysis results indicating no significant difference.

Some potential limitations of this study should be addressed. First, the FAERS data (a self-reporting database) have some inherent limitations. FAERS does not require a causal relationship between the product and the event to be proven, and reports do not always contain enough detail to properly evaluate an event. Additionally, the data might contain inaccurate and incomplete information, such as age and countries of occurrence^34^. Fortunately, in our study, the missing rate for the countries of occurrence used in the subgroup disproportionality analysis was low, at 1.6%. However, there were many cases of missing data for indication of sacubitril/valsartan or ARBs used in the sensitivity analysis. Second, the duration of drug use and the time between drug administration and AE occurrence were not considered in the analysis. The accumulation of amyloid-β caused by sacubitril and the development of cognitive impairment may take several decades^4,35^. Differences in the duration of sacubitril usage across continents and individuals were not taken into account. Third, since ARBs are a relatively old class of drugs, the reporting frequency of associated AEs tends to be lower than the actual occurrence of AEs. The known adverse effects of drugs and their quantification, therefore, tend to be underestimated. Fourth, the list of ARBs that we have set does not include combination drugs containing ARBs. However, to assess the precise association of the AEs, it was essential to exclude combination formulations containing components other than ARBs. Fifth, the definition of dementia is heterogeneous and relies solely on declarative data. Lastly, while our study was conducted on a continent-specific basis, it is essential to consider the diverse range of ethnicities encompassed within each continent. However, due to the unavailability of data on specific ethnic groups, the categorization by continent was primarily employed as a surrogate measure for assessing pharmaco-ethnic vulnerability^19^. Notwithstanding these limitations, this study is significant as the first to analyze data regionally.

There are regional variations in reporting of dementia-related AEs associated with sacubitril/valsartan. Specifically, contrary to previous studies, a ROR value greater than 1 was observed in North America. Despite limitations in FAERS data, the elevated ROR values identified in North America suggest caution may be warranted regarding potential dementia-related AEs associated with sacubitril/valsartan. Further research, including long-term follow-up, is warranted in North America to investigate the association between sacubitril/valsartan and dementia-related AEs.

## Supplementary Information

Supplementary Table S1.

Supplementary Table S2.

Supplementary Table S3.

Supplementary Table S4.

## Data Availability

The data that support the findings of this study are available from the corresponding author upon reasonable request.
