# Supplementary material for: Subgroup disproportionality analysis of dementia-related adverse events with sacubitril/valsartan across geographical regions
Source: Sci Rep. 2024 Sep 3;14:16408. doi: 10.1038/s41598-024-67050-5 (PMC11372112; doi:10.1038/s41598-024-67050-5)
Supplement: Supplementary file 1 — Supplementary Table S1. [file 41598_2024_67050_MOESM1_ESM.docx]

**Table S1.** 2×2 contingency table for calculating reporting odds ratios

|  | Sacubitril/valsartan | ARBs |
| --- | --- | --- |
| Dementia-related AEs | A | B |
| Other AEs | C | D |

AE, adverse event; ARB, angiotensin receptor blocker
