# Supplementary material for: Subgroup disproportionality analysis of dementia-related adverse events with sacubitril/valsartan across geographical regions
Source: Sci Rep. 2024 Sep 3;14:16408. doi: 10.1038/s41598-024-67050-5 (PMC11372112; doi:10.1038/s41598-024-67050-5)
Supplement: Supplementary file 2 — Supplementary Table S2. [file 41598_2024_67050_MOESM2_ESM.docx]

**Table S2.** Preferred terms (PTs) related to dementia (Broad SMQ)

| “abnormal behaviour”, “abulia”, “activities of daily living impaired”, “affect lability”, “aggression”, “feeling abnormal”, “agitation”, “agnosia”, “amnesia”, “amnestic disorder”, “anterograde amnesia”, “learning disorder”, “apathy”, “hostility”, “aphasia”, “apraxia”, “borderline mental impairment”, “cerebral atrophy”, “symbolic dysfunction”, “cerebral atrophy congenital”, “change in sustained attention”, “cognitive disorder”, “confusional state”, “delirium”, “morose”, “delusion”, “delusional disorder, jealous type”, “delusional disorder, unspecified type”, “disinhibition”, “memory impairment”, “disorientation”, “disturbance in social behaviour”, “executive dysfunction”, “flat affect”, “hallucination”, “hypomania”, “illusion”, “impaired reasoning”, “inappropriate affect”, “initial insomnia”, “intelligence test abnormal”, “irritability postvaccinal”, “judgment impaired”, “learning disability”, “mental status changes”, “mood altered”, “mood swings”, “negativism”, “neuropsychological test abnormal”, “sexually inappropriate behaviour”, “personality change”, “prodromal alzheimer’s disease”, “psychotic behaviour”, “psychotic disorder”, “restlessness”, “social avoidant behaviour”, “somnambulism”, “somnolence”, “sopor”, “speech disorder”, “suspiciousness”, “thinking abnormal”, “transient global amnesia”, “vascular cognitive impairment”. |
| --- |
