# Supplementary material for: Subgroup disproportionality analysis of dementia-related adverse events with sacubitril/valsartan across geographical regions
Source: Sci Rep. 2024 Sep 3;14:16408. doi: 10.1038/s41598-024-67050-5 (PMC11372112; doi:10.1038/s41598-024-67050-5)
Supplement: Supplementary file 3 — Supplementary Table S3. [file 41598_2024_67050_MOESM3_ESM.docx]

**Table S3.** Preferred terms (PTs) related to heart failure

| “Acute left ventricular failure”, “Acute pulmonary oedema”, “Acute right ventricular failure”, “Cardiac asthma”, “Cardiac failure”, “Cardiac failure acute”, “Cardiac failure chronic”, “Cardiac failure congestive”, “Cardiac failure high output”, “Cardiogenic shock”, “Cardiohepatic syndrome”, “Cardiopulmonary failure”, “Cardiorenal syndrome”, “Chronic left ventricular failure”, “Chronic right ventricular failure”, “Cor pulmonale”, “Cor pulmonale acute”, “Cor pulmonale chronic”, “Ejection fraction decreased”, “Hepatic congestion”, “Hepatojugular reflux”, “Left ventricular failure”, “Low cardiac output syndrome”, “Neonatal cardiac failure”, “Obstructive shock”, “Pulmonary oedema”, “Pulmonary oedema neonatal”, “Radiation associated cardiac failure”, “Right ventricular ejection fraction decreased”, “Right ventricular failure”, “Ventricular failure”, “Artificial heart implant”, “Atrial natriuretic peptide abnormal”, “Atrial natriuretic peptide increased”, “Bendopnoea”, “Brain natriuretic peptide abnormal”, “Brain natriuretic peptide increased”, “Cardiac cirrhosis”, “Cardiac contractility modulation therapy”, “Cardiac device reprogramming”, “Cardiac dysfunction”, “Cardiac index decreased”, “Cardiac output decreased”, “Cardiac resynchronisation therapy”, “Cardiac ventriculogram abnormal”, “Cardiac ventriculogram left abnormal”, “Cardiac ventriculogram right abnormal”, “Cardiomegaly”, “Cardio-respiratory distress”, “Cardiothoracic ratio increased”, “Central venous pressure increased”, “Diastolic dysfunction”, “Dilatation ventricular”, “Dyspnoea paroxysmal nocturnal”, “Heart transplant”, “Hepatic vein dilatation”, “Implantable cardiac monitor replacement”, “Intracardiac pressure increased”, “Jugular vein distension”, “Left ventricular diastolic collapse”, “Left ventricular dilatation”, “Left ventricular dysfunction”, “Left ventricular enlargement”, “Lower respiratory tract congestion”, “Myocardial depression”, “Nocturnal dyspnoea”, “N-terminal prohormone brain natriuretic peptide abnormal”, “N-terminal prohormone brain natriuretic peptide increased”, “Oedema”, “Oedema blister”, “Oedema due to cardiac disease”, “Oedema neonatal”, “Oedema peripheral”, “Orthopnoea”, “Peripheral oedema neonatal”, “Peripheral swelling”, “Post cardiac arrest syndrome”, “Prohormone brain natriuretic peptide abnormal”, “Prohormone brain natriuretic peptide increased”, “Pulmonary congestion”, “Right ventricular diastolic collapse”, “Right ventricular dilatation”, “Right ventricular dysfunction”, “Right ventricular enlargement”, “Scan myocardial perfusion abnormal”, “Stroke volume decreased”, “Surgical ventricular restoration”, “Systolic dysfunction”, “Venous pressure increased”, “Venous pressure jugular abnormal”, “Venous pressure jugular increased”, “Ventricular assist device insertion”, “Ventricular compliance decreased”, “Ventricular dysfunction”, “Ventricular dyssynchrony”, “Wall motion score index abnormal”) |
| --- |
