# Supplementary material for: Subgroup disproportionality analysis of dementia-related adverse events with sacubitril/valsartan across geographical regions
Source: Sci Rep. 2024 Sep 3;14:16408. doi: 10.1038/s41598-024-67050-5 (PMC11372112; doi:10.1038/s41598-024-67050-5)
Supplement: Supplementary file 4 — Supplementary Table S4. [file 41598_2024_67050_MOESM4_ESM.docx]

**Table S4.** Characteristics of adverse event reports by continent

|  | Asia  (n = 2,355) | Africa  (n = 410) | Europe  (n = 7,249) | North America  (n = 9,465) | Latin America  (n = 1,835) | Oceania  (n= 195) |
| --- | --- | --- | --- | --- | --- | --- |
| Age (year), median (Q1–Q3) | 72 (66–79) | 70 (65–77) | 75 (68–82) | 71 (65–78) | 75 (68–83) | 75 (69–83) |
| Sex, n (%) |  |  |  |  |  |  |
| Male | 1,515 (64.3) | 246 (60.0) | 3,531 (48.7) | 5,075 (53.6) | 854 (46.5) | 117 (60.0) |
| Female | 798 (33.9) | 164 (40.0) | 3,629 (50.1) | 4,079 (43.1) | 966 (52.6) | 74 (37.9) |
| Missing | 42 (1.8) | 0 (0.0) | 89 (1.2) | 311 (3.3) | 15 (0.8) | 4 (2.1) |
| Reporter, n (%) |  |  |  |  |  |  |
| Physician | 690 (29.3) | 113 (27.6) | 2,756 (38.0) | 1,528 (16.1) | 190 (10.4) | 83 (42.6) |
| Pharmacist | 84 (3.6) | 10 (2.4) | 1,066 (14.7) | 527 (5.6) | 7 (0.4) | 4 (2.1) |
| Other health-professional | 491 (20.8) | 42 (10.2) | 2,197 (30.3) | 1,660 (17.5) | 253 (13.8) | 79 (40.5) |
| Consumer/lawyer | 1,076 (45.7) | 243 (59.3) | 1,090 (15.0) | 5,684 (60.1) | 1,365 (74.4) | 29 (14.9) |
| Missing | 14 (0.6) | 2 (0.3) | 140 (1.9) | 66 (0.7) | 20 (1.1) | 0 (0.0) |
